# How to optimize knowledge construction in the brain

**DOI:** 10.1038/s41539-020-0064-y

**Published:** 2020-05-01

**Authors:** Marlieke Tina Renée van Kesteren, Martijn Meeter

**Affiliations:** 1 Section of Education Sciences, Vrije Universiteit Amsterdam, Amsterdam, The Netherlands; 2 Institute of Brain and Behavior Amsterdam, Vrije Universiteit Amsterdam, Amsterdam, The Netherlands; 3 LEARN! Research Institute, Vrije Universiteit Amsterdam, Amsterdam, The Netherlands

**Keywords:** Long-term memory, Human behaviour

## Abstract

Well-structured knowledge allows us to quickly understand the world around us and make informed decisions to adequately control behavior. Knowledge structures, or schemas, are presumed to aid memory encoding and consolidation of new experiences so we cannot only remember the past, but also guide behavior in the present and predict the future. However, very strong schemas can also lead to unwanted side effects such as false memories and misconceptions. To overcome this overreliance on a schema, we should aim to create robust schemas that are on the one hand strong enough to help to remember and predict, but also malleable enough to avoid such undesirable side effects. This raises the question as to whether there are ways to deliberately influence knowledge construction processes, with the goal to reach such optimally balanced schemas. Here, we will discuss how the mnemonic processes in our brains build long-term knowledge and, more specifically, how different phases of memory formation (encoding, consolidation, retrieval, and reconsolidation) contribute to this schema build-up. We finally provide ways how to best keep a balance between generalized semantic and detailed episodic memories, which can prove very useful in, e.g., educational settings.

## Introduction

Our brains are optimized to remember important information to various degrees. At the same time, they have to be able to quickly discard irrelevant, non-repetitive details^1^. By forming knowledge structures, or schemas, new information that fits prior knowledge can allegedly be encoded more efficiently than when information is novel^2,3^. Moreover, post-encoding processes such as consolidation and retrieval are presumed to be facilitated once new experiences fit with previously encountered experiences^4,5^. New information then becomes integrated with existing schemas rather than independently stored^2^. However, recent findings in cognitive neuroscience^6^, as well as long-standing findings in psychology^7^ and educational science^8^, suggest that this generalization process has a downside. That is, when a schema becomes too strong, it can erroneously associate new experiences. This process can facilitate the formation of false memories and misconceptions^9^.

Here, we will discuss this paradox from a cognitive neuroscience viewpoint, and propose ways to optimally build and use schemas while simultaneously avoiding schema-overreliance. We consider and link different memory types and phases of memory formation (encoding, consolidation, reconsolidation, and retrieval), and review how these contribute to schema construction. We also focus on possible functions of these neural processes by highlighting the roles of predictive coding^10,11^ and place and grid cells^12^ in the hippocampus, as well as interactions between the hippocampus and other brain structures such as the (ventro)medial prefrontal cortex (mPFC)^13^. Then, we describe possible ways to actively and passively influence knowledge construction such that we can flexibly adapt and shape emerging schemas, e.g., through considering the neural processes underlying encoding, reactivation^14,15^, consolidation^4,16^, and memory integration^17^.

**Fig. 1:**
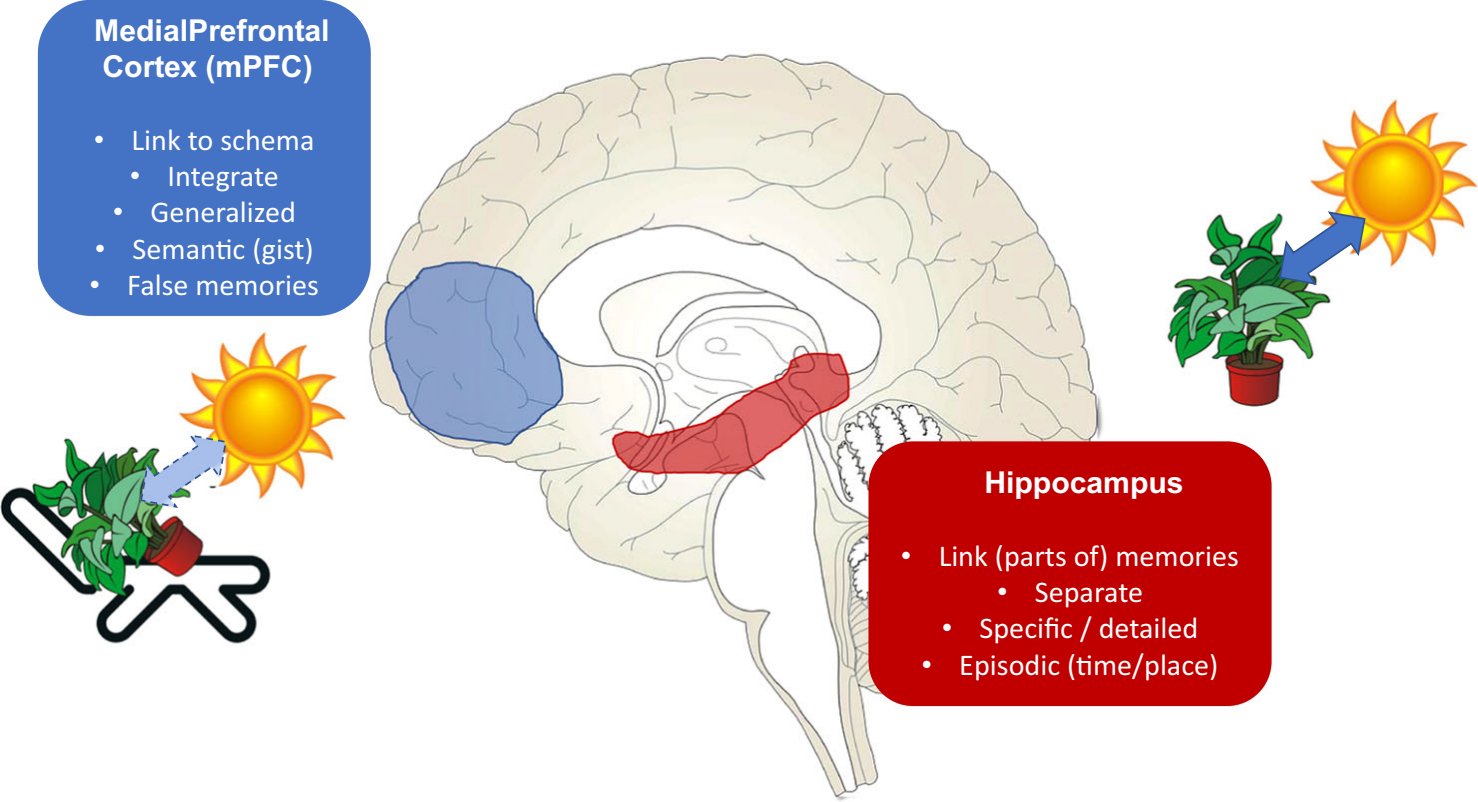
The hippocampus and mPFC are presumed to have different functions when it comes to storing memories. Where the hippocampus is suggested to link separate parts of a memory into specific, detailed episodic memories, the mPFC is proposed to integrate memories into existing knowledge schemas while inhibiting the hippocampus, leading to a generalized, semantic memory. Both these processes are highly valuable to long-term memory formation. However, the integrative process governed by the mPFC, along with the semanticization processes during consolidation, can also lead to false memories or misconceptions (see example in the main text where the necessary effect of sunlight on plant survival can be misinterpreted because it is not vital, just pleasant, for humans). All images are rights-free and the bed icon is used with permission from http://www.toicon.com/.

Finally, we will reflect on possible future steps in knowledge construction research, as well as applications for situations where such processes plays a pivotal role, such as in educational settings^14^. We will revisit commonly used study techniques like retrieval practice^5^, spaced learning^18^, and desirable difficulties^19^ and place them in a neural context. Also, we discuss possible means to avoid the emergence of false memories and misconceptions, which signify a major burden for learning in educational situations. By doing so, we aim to provide guidelines from a neural perspective that can provide a basis for novel research and can additionally be used to achieve optimal knowledge acquisition practices. We here chose to focus on the adult brain, but acknowledge that not all we discuss might translate directly to the developing brain^20^.

## The phases of memory

The formation of long-term declarative memories is proposed to be governed by a set of successive processes in the brain. First, new information becomes encoded into a memory by the hippocampus and surrounding regions in the medial temporal lobe (MTL)^21^. The hippocampus is thought to connect different parts of a memory that make up a specific episode. So right after encoding, a memory is usually episodically detailed, containing details about time and place^22^. Over time, encoded memories are proposed to become consolidated during sleep^4,23^ (and presumably also during rest^24–27^). The specifics of this process are still up to debate, but it is generally argued that consolidation acts to semanticize memories so that episodic details fade away and a semantic network of separately acquired, associated memories remains^28,29^. According to the systems consolidation model^30^, this leads a memory to become hippocampally independent after some time, meaning that the hippocampus is not necessary anymore to access the memory.

An alternative theory, multiple trace or transformation theory^31–33^ poses that this process transforms memories such that the memory will contain episodic details only when the hippocampus can still access the memory. Otherwise, a memory can still be expressed, but will contain few or no episodic details. Generally, when memories are retrieved^34^ after consolidation, they have a different neural architecture than when they were first encoded^4^. Moreover, they do not always contain the same details, showing that memories are altered over time beyond our conscious awareness and are not retrieved as a solid entity, but rather reconstructed depending on present cues^35,36^. This also means that one memory can consist of both episodic and semantic features depending on how it is retrieved.

The memory cycle does not finish there^28^. The act of retrieval is generally thought to alter a memory again, updating it with previously and currently learned or retrieved information. Memories are then suggested to become reconsolidated into existing schemas, presumably altering their features again^16,37^. This way, schemas are thought to be continuously adjusted to optimize our understanding of the world around us and to allow prediction of future occurrences^38^.

## Knowledge building and schemas

Already in the early 1900’s, Fredric Bartlett coined the term schema to denote “a structure that people use to organize current knowledge and provide a framework for future understanding”^39^. Similarly, in educational psychology, Jean Piaget used the term schema to explain how young kids learn regularities in the environment^40^. Piaget’s concepts regarding accommodation (the adaptation of an existing schema) and assimilation (integration of new information into a schema) are still prevalent throughout educational theory, most importantly in constructivism^41^. Over the years, schemas have been investigated in different cognitive research areas but only appeared within cognitive neuroscience after a seminal paper by the Richard Morris lab in 2007^42^. Here, rats were found to more quickly encode and consolidate new information when they fitted with a spatial schema^42,43^. Since then, a multitude of research has been published, both in animals and humans, that focuses on the question how the brain constructs, uses, and adapts schemas and what this means for how our memories are stored^3^.

In this past decade, many theories regarding the nature of schematic knowledge in our brains have been proposed. Here, we will focus specifically on human, whole-brain, and systems-level theories. Most of these discuss the roles of brain regions such as the hippocampus and the mPFC and their relationship towards storing, accessing and updating schemas. Overall, it has been proposed that the mPFC plays a role in accessing the schema in order to update it^2,3,44,45^. More specifically, the SLIMM-framework predicts that the mPFC detects resonance from an activated schema and then acts to directly integrate newly learned information^2^ a process that is usually achieved through extensive offline processes such as (re)consolidation^37^. Such an integration state is likely dissimilar to encoding and retrieval states^46,47^. The hippocampus has been suggested to update schemas containing spatially-oriented information^48–50^, but the SLIMM-framework proposes that the hippocampus is not related to integrating new information with existing schemas^2^. In fact, it is suggested to be inhibited by the mPFC in such situations, hence explaining differential connectivity patterns between these regions.

Overall, we can carefully conclude that the brain can store information in multiple ways, and the hippocampus and mPFC represent two important brain regions underlying this process. These regions are assumed to determine whether a memory gets stored with few or many episodic details and with weak or strong connections to an existing schema. Knowing how to actively recruit these regions can potentially help us to store memories in a desired way.

## The predictive brain

To understand how and why the brain organizes information into schemas, it is important to also consider theories about brain functioning. For example, theories about how memories are shaped through, e.g., consolidation processes can give insight as to why our brain organizes information the way it does^29,32^. The brain has evolved to optimally survive in the world and memory plays an important part in survival. However, it is not always useful to remember only unique episodic events. For example, to know where to best find nuts and berries, you can remember a specific place where they are found each year, but you could also extract a general rule that predicts where they usually grow. This hierarchical memory system, moving from a very specific memory containing time and place-specific details to generalizations that predicts how the world is organized, is proposed to depend on selection processes during consolidation^4,51^. This selection is crucial to survive in a dependable but yet ever-changing environment. According to a theory posed by Buzsáki and Moser, the place and grid cell organization in the MTL that explains how we can flexibly navigate our surroundings, is highly suited to support such a hierarchical memory system^52^.

Such thinking fits well with predictive coding^53^, a general theory of brain function stating that our brains evolved to predict what will happen next. To do so, the brain needs a clear and consistent world model. This model is similar to semantic memory or schema, and is suggested to generate prediction errors^54^ when inconsistent information is encountered^55^. Moreover, it can optimize mnemonic processing through prospection^56^ recruiting, among others, the mPFC^38^. This works well when the model is not yet fully developed. However, when it becomes very strong it will be resistant to change; many and large prediction errors are needed to adapt it.

Accordingly, it appears that the brain hosts different ways to form a memory and create knowledge schemas. Our brain is continuously absorbing information from the environment to optimize its internal predictive model. Conversely, detailed episodic memories are also valuable as they can help to update the model when the world happens to change. How can we optimally utilize this neural architecture to create memories that contribute to a malleable but durable schema while preserving relevant details and avoiding the formation of erroneous, but well-fitting memories?

## False memories

The need for such a middle road is exemplified by an undesirable consequence of such predictive processes in our brains: They can give rise to false memories^7^. The precise definition of a false memory is debated^57^ and shows strong overlap with misinformation^58^, misattribution^59,60^ and misconception^8^ effects. False memories are shown to become “implanted”^9^, inferred^7^, or distorted^35^ through presenting participants with either wrong, incomplete, or overlapping information. For example, Elizabeth Loftus has made her participants remember memories that never existed, such that they were lost in a shopping mall as a child^61^. Moreover, in the Deese–Roediger–McDermott (DRM) paradigm^7^, participants that learn a list of words such as “snow”, “cold”, and “dark” will later report that the list contained the associated lure “winter”. Finally, in a related reconsolidation paradigm, participants who learned two word lists were found to erroneously assign words from the second to the first list^60^. This happened particularly after a short reminder, which presumably led to integration of the two separately learned sets of information^62^.

The neural processes underlying such false memory formation have mostly been attributed to the reconstructive nature of retrieval processes^35^, especially when reactivating previous memories during new learning^6,59,63^. Brain regions such as the (m)PFC, semantic relatedness regions, and reinstatement effects in item-specific areas appear to allow the formation of false memories^6,59,64,65^. Moreover, the mPFC has been found to relate to misattribution in the DRM-paradigm, such that patients with mPFC-lesions do not show false recall effects^66^. This finding, together with other (virtual) lesion studies on schema effects^67–69^, suggests that schema-related integratory mechanisms are important when generating false memories.

## How can we best make memories?

From the above, we can distill that the definition of a good memory is not clear-cut, let alone how to create such a memory. Usually, we think of a good memory as vivid, strong, and episodically detailed, such as memories of important life events (like graduation ceremonies). This is an important feature of autobiographical memories^70^. Yet, such vividness might not necessarily be important in any situation. In education, a good memory is usually detailed (e.g., which elements enable photosynthesis in plants), but does not need to contain episodic details such as when and where you first learned it. Alternatively, a valuable memory also allows you to see the bigger picture so that you can, e.g., infer that sunlight is essential for plants as without it they will not be able to create vital sugars and will die (Fig. 1). At the same time, students should not generalize this process to humans, thinking that sunlight is a pleasant but not required factor in a plant’s life, because for us humans the sun is not necessary for our survival either (at least not in the short term). To get rid of this misconception, students can, e.g., go back to the initial details relating to photosynthesis and revisit its components. They could elaborate on why sunlight works differently for plants than for animals and come up with detailed stories or examples. This way, their schema can be updated so they will no longer confuse plant physiology with animal physiology.

Such a dynamic memory^71,72^ is extremely valuable. Students should therefore strive to create memories containing both episodic and semantic features. A perfect memory benefits from belonging to an overarching schema, but should not yet be fully semanticized such that all distinguishing details have disappeared^73^ and false memories can arise. With this in mind, we will now highlight some ways in which insights from cognitive (neuroscience) research can provide guidelines to optimize dynamic memory formation during different memory phases (also see Box 1).

### Encoding

During memory encoding there are several tricks to facilitate long-term retention of (detailed) information. Here, we will highlight the most important. First, elaboration helps to relate a new memory to as many existing traces as possible, thereby presumably facilitating integration with prior knowledge^74^. This technique can be practiced in multiple ways. For example, you can ask yourself questions about what you already know about a topic or how it links to other topics. Moreover, the well-established method of loci extends new memories by linking them to spatial locations and combining different sensory systems to make the memory as vivid as possible^75^. This method can be easily trained and is shown to change neural processes underlying memory formation^76,77^.

Similarly, reactivation of previous memories while learning new information is suggested to help to integrate them with an existing schema^78^, presumably making a new memory better connected and less likely to fade away^17^. All of this should happen at a desirable difficulty^79^, at an optimal “distance” from the schema. This is rooted in the assumption that when new information is too alike you will not learn a lot, but very distinct information cannot be linked well either.

Distinctiveness itself is also a characteristic that makes a memory more easily retrievable. It has long been known that events that stand out from their context are more easily remembered^80^. This has been viewed as an effect of novelty enhancing encoding, but it probably reflects easier recall of distinctive memories^81,82^. Making one item stand out from its context, e.g., by printing a word in a text in a different font, does not increase the strength with which it is encoded. However, it makes the word distinctive, which makes it easier to retrieve from memory (due to lessened interference). This was shown, for example, by a study in which items became distinctive only after they had been studied, i.e., by manipulating subsequent items, distinctiveness still led to better retrieval, even though it could not have affected the encoding of the items^81^. Novelty does affect encoding, though, but not on an item by item basis. Several studies have found that exploring a novel environment^83,84^ or seeing a set of novel pictures^85^ enhances encoding of subsequently learned material, up to thirty minutes later. This suggests that novelty can create a time window for strong learning in your hippocampus^55^, probably mediated through release of dopamine^86^.

A new avenue for research into encoding enhancement is that of brain stimulation^87^, either through non-invasive methods like transcranial magnetic stimulation (TMS) or transcranial direct current stimulation (tDCS), or by invasively stimulating brain regions with intracranial electrodes (deep brain stimulation; DBS)^88^. Up to now, effects are very mixed, it is uncertain which regions can best be targeted and how stimulation spreads through the brain^87^. Therefore, this research area needs more work before being able to be applied in daily practice.

Box 1: Educational applications In daily life, we can use above-mentioned techniques to facilitate learning and long-term memory formation. In general, it appears that encoding and retrieval are processes during which we can and should focus on checking our schema and adding episodic details. Conversely, memory consolidation is a process during which we, mostly unconsciously, extract commonalities and expand schemas, often at the cost of specific details. So, in order to ensure a good balance between semantic and episodic memories in educational settings, we can follow these tricks:

1. Elaborate where you can, both during encoding and retrieval. Use a wide range of knowledge and senses to make a memory as vivid as possible, yet also connected to prior knowledge. Considering how the hippocampus uses spatial properties to learn, e.g., by using the method of loci, can help.
2. Reactivate prior knowledge when you learn new information, not only to connect old and new information, but also to be able to apply retrieval practice strategies to strengthen already existing knowledge and find links between newly learned information and existing knowledge. This way, you can best find a balance between memory for details and gist knowledge.
3. Use breaks wisely. Space and interleave your studying and repeat, most optimally through retrieval, information on separate days. This allows you to accommodate spacing and consolidation effects that help you to semanticize information and build strong schemas.
4. Keep track of detail loss during retrieval. It is often important to remember details, especially in educational situations. In such cases, you can keep a list of important details (e.g., years, numbers, names etc.) and study these separately. Or reactivate them when you learn new information (see point 2) so you can create a new detailed episodic memory.
5. Spot false memories and misconceptions. Whenever you notice that your extensive, but semanticized knowledge leads you to infer misconceptions or lose details, revert to point 1 and point 4 to override them. Discovery of such misconceptions can be achieved by incorporating regular checks, either by yourself or by others.

### Consolidation

After encoding, during online and offline consolidation, memories continue to be adapted. As a result their neural signatures can become more pronounced^89^ or more alike similar memories^90^. This is arguably one of the reasons why spacing^91^ and interleaving^92^ of topics over time have pronounced effects on memory performance^93^. Through spacing, information is rehearsed at set times, hours, days or even weeks apart. Interleaving involves interchangeably studying different types of information. Sleep is the most important means to consolidate, integrate and semanticize memories, which is supposed to generalize memories^4^, foster insight^94^, and create strong schemas^29,90^. In addition, offline rest periods, in which participants are told to just let their mind wander, show consolidation-like effects on memory performance as well^25–27^. In general, letting memories “rest” and coming back to them on a regular basis is considered a good tactic to make strong schemas.

Such consolidation processes can be optimized to allow for enhanced learning and remembering^95^. First and foremost, you should aim to get enough sleep per night, especially after you studied new information^95^. Regular breaks between studying, optimally periods in which little new information is absorbed by the brain, are presumed to be beneficial for ongoing mnemonic processing^96^. Take a walk, read a simple book or magazine, or allow yourself a short nap or rest. Moreover, adding (emotional) value to to-be-learned information^97,98^, or actively repeating information through auditory presentation during sleep (targeted memory reactivation; TMR)^99,100^, has been shown to help strengthen memories. Unfortunately, such effects are small and specific and hence not easy to practice in daily life.

### Retrieval

Retrieval of stored memories, as mentioned above, is not a passive process. It can initiate reconsolidation^16,37^ processes that further modify and integrate a memory with currently available information. Moreover, iterative associative retrieval is shown to yield neural signatures related to both generalization in the mPFC and episodic specificity in parietal regions^101^. This is probably a reason why retrieval practice is so effective in educational situations^102,103^. It allows to strengthen a memory through further elaboration^104^ and integration with other activated knowledge, perhaps akin to consolidation processes during sleep^5^. Retrieval practice can be applied in educational settings in many ways, through, e.g., regular quizzes^105^, teaching one another^106^, or flipping the classroom (i.e., having students study content at home and (collectively) making homework assignments in class)^107^.

Another way to actively enhance retrieval of details is through episodic specificity induction^108^, in which participants are trained^109^ to recall previously learned information with high episodic detail. This method has been shown to, e.g., enhance detail generation^110^, imagination of future events^111^, and creativity^112^, and recruits episodic memory brain regions such as the hippocampus^111^. Unfortunately, however, false memory formation (i.e., sensitivity to critical lures in the DRM-paradigm) is also enhanced through this method^113^. As a result, further research is necessary to find out whether and how such methods can be applied in educational contexts.

Finally, a method that can presumably be applied to all memory phases is that of entrainment of brain oscillations^114^. The idea behind this method is that brain oscillations that are beneficial for memory can be modulated through presentation of visual or auditory “flickers” in the same frequency or through DBS. Just like stimulation techniques, this technique is still very unspecific and needs further research before it can be applied.

## Future research

Understanding how the memory mechanisms in our brain work can potentially help to optimize our memories. Here, we described that memory processes happen on different levels and at different time scales. Moreover, we showed that memories can exist in different dimensions, along a continuum of episodic-semantic, ranging from very detailed memories to general knowledge schemas. This knowledge can be useful in understanding how to provide a perfect balance between episodic details and links to schemas. Future research should therefore consider and examine ways to best achieve this balance, guided by educational and psychological insights into memory enhancement techniques in combination with neuroscientific knowledge. Importantly, as we acknowledged at the start of this paper but have not been able to cover, the development of the brain should also be taken into account, as the regions implicated above do function differently over development^115,116^. Also, the interaction between the mPFC and the MTL in storing different types of memories is a research area that requires further attention.

For instance, we can look for (metacognitive) measures, or develop paradigms to detect the interplay between the effects of a schema on the expression of a specific episodic memory^72^. We can then track schema building processes over time and find out how to best “episodicize” an already existing schema. This might on the one hand help to avoid or recognize overly generalized memories that cause misconceptions. Alternatively, tracking the amount of detailed knowledge at regular intervals can account for forgetting of important details, which is important in many aspects of daily life.

## Conclusions

Here we provided an overview of the current literature related to memory processes, theories, and enhancement strategies at different periods in the lifetime of a memory. We have shown that, over time, memories “semanticize” into an overarching schema, which leads them to be stronger and less likely to be forgotten. We value such schemas and want to facilitate their construction. However, partly through this process, episodic details often fade away and might be forgotten. Moreover, false memories are more likely to arise with very strong schemas. Such side effects are unwanted, especially in educational settings where we strive for a balance between episodic and semantic features. We therefore ended our review with some preliminary tips on how to reach this balance, and provided avenues for future research into this topic.
